# New species of *Asphalidesmus* Silvestri, 1910 from Australia (Diplopoda, Polydesmida, Dalodesmidea)

**DOI:** 10.3897/zookeys.93.1255

**Published:** 2011-04-29

**Authors:** Robert Mesibov

**Affiliations:** Queen Victoria Museum and Art Gallery, Launceston, Tasmania, Australia 7250

**Keywords:** millipede, New South Wales, Queensland, Tasmania, Victoria

## Abstract

*Asphalidesmus allynensis* **sp. n.** and *Asphalidesmus dorrigensis* **sp. n.** are described from New South Wales, *Asphalidesmus otwayensis* **sp. n.** from Victoria, and *Asphalidesmus bellendenkerensis* **sp. n.**, *Asphalidesmus carbinensis* **sp. n.**, *Asphalidesmus magnus* sp. n. and *Asphalidesmus minor* **sp. n.** from Queensland. The previously endemic Tasmanian genus *Asphalidesmus* Silvestri, 1910 is now known from 16°S to 43°S in eastern Australia, a north-south range of ca 3000 km. *Asphalidesmus* spp. throughout this range are very similar in overall appearance. Three of the new species are able to coil in a tight spiral.

## Introduction

**Figure 1.**
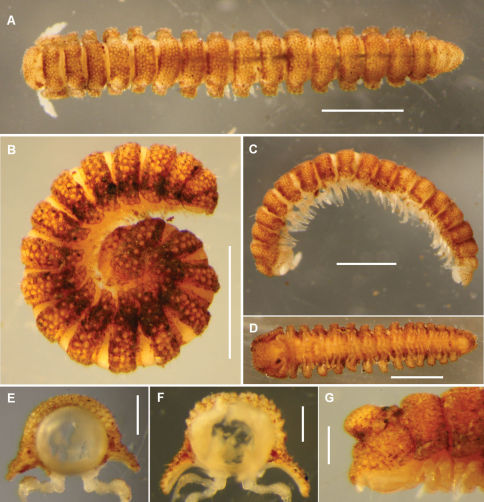
**A** *Asphalidesmus leae* Silvestri, 1910, dorsal view of female, QVM 23:52009 **B** *Atopodesmus dorrigensis* sp. n., lateral view of coiled adult paratype, AM KS61085 **C** *Atopodesmus leae* Silvestri, 1910, lateral view of curled male, QVM 23:52009 **D** *Atopodesmus magnus* sp. n., ventral view of male, ANIC 64-000204 **E, F** Anterior views of ring 6 of males of *Atopodesmus leae* Silvestri, 1910 (**E**, QVM 23:41547) and paratype *Atopodesmus magnus* sp. n. (**F**, QM S90026) **G** *Atopodesmus otwayensis* sp. n., lateral view of last rings of paratype female, ANIC 64-000207. Scale bars: **A–D** = 1 mm, **E–G** = 0.25 mm.

*Asphalidesmus* Silvestri, 1910 is a genus of tiny, gregarious, slow-moving Polydesmida living in moist leaf litter and rotting logs. Juveniles are pure white in colour, while adults are yellow-brown (Fig. 1) and often encrusted with soil particles. The genus is currently placed in the suborder Dalodesmidea Hoffman, 1980 but has not yet been assigned to a family (Golovatch 2003, Mesibov 2009).

The three previously described species of *Asphalidesmus* are endemic to Tasmania (Mesibov 2002, 2009), where they occur in rainforest and wet eucalypt forest, and sometimes in caves. In this paper I describe seven new species of *Asphalidesmus* from the Australian mainland. The genus is now known to range from 16°03'S in tropical northern Queensland to 43°28'S in cool temperate southern Tasmania, or ca 3000 km north-south.

There are very wide gaps in the currently known distribution of *Asphalidesmus* in eastern Australia (Fig. 12). The gaps contain blocks of rainforest and wet eucalypt forest, and it is likely that some of those forests are home to undiscovered species of these cryptic millipedes. However, I have repeatedly searched for litter- and log-dwelling millipedes in the forested, high-rainfall Strzelecki Ranges in southeastern Victoria (Mesibov 2007 and unpublished observations) and have not collected an *Asphalidesmus* species there. It is also interesting that I recovered *Asphalidesmus* specimens from berlesates from northern Queensland rainforest, but not from rainforest in southeastern Queensland. The two regions have been intensively sampled by the same group of fieldworkers using the same methods, with roughly the same totals of collecting events (Fig. 3 in Mesibov 2008).

*Asphalidesmus* females from three eastern Australian sites remain unidentified because no males have yet been collected at those or nearby locations. The sites are shown on the distribution map (Fig. 12) and the females listed in the Appendix as ‘*Asphalidesmus* sp.’

## Methods

‘Male’ and ‘female’ in the text refer to adult, stadium 7 individuals. All specimens are stored in 75–80% ethanol in their respective repositories.

Gonopods were cleared and temporarily mounted in 60% lactic acid for optical microscopy. Preliminary drawings on graph paper were made using an eyepiece grid at 160X on a binocular microscope. Photomicrographs were taken with a Canon EOS 1000D digital SLR camera mounted on a Nikon SMZ800 binocular dissecting microscope equipped with a beam splitter. Measurements were made with the same microscope using an eyepiece scale. Specimens for scanning electron microscopy were air-dried, sputter-coated with platinum and examined with a Hitachi SU-70 operated in high-vacuum mode.

All images and drawings were prepared for publication using GIMP 2.6. Focus-stacking was used to add depth of field to some photomicrographs. Maps were generated using ArcView GIS 3.2.

A list of all currently known localities for all *Asphalidesmus* species is given in the Appendix. Locality details there and in the text below are given in all cases with latitude and longitude based on the WGS84 datum. I also estimate a conservative uncertainty for each locality, expressed in metres as the radius of a circle around the stated position. Latitude and longitude data given below in italics are based on museum collection databases, maps or Google Earth, as indicated in the Remarks sections.

Abbreviations: AM = Australian Museum, Sydney, NSW; ANIC = Australian National Insect Collection, Canberra, Australian Capital Territory; MV = Museum Victoria, Melbourne, Vic; NSW = New South Wales; Qld = Queensland; QM = Queensland Museum, Brisbane, Qld; QVM = Queen Victoria Museum and Art Gallery, Launceston, Tasmania; Vic = Victoria.

## Results

**Order Polydesmida Pocock, 1887**

**Suborder Dalodesmidea Hoffman, 1980**

### Asphalidesmus

Silvestri, 1910

http://species-id.net/wiki/Asphalidesmus

Asphalidesmus Silvestri 1910: 362. Attems 1914: 242, 1926: 153, 1931: 77, 1940: 205. Brölemann 1916: 547. Verhoeff 1932: 1587, 1936: 12. Jeekel 1971: 313, 1982: 12, 1984: 85, 1986: 46. Hoffman 1980: 150. Mesibov 2002: 532, 2009: 67. Golovatch 2003: 53. Golovatch et al. 2009: 3. Mesibov 2009: 67.

#### Type species:

*Asphalidesmus leae* Silvestri, 1910, by original designation.

*Atopodesmus* Chamberlin 1920: 153. Attems 1926: 134, 1940: 356. Verhoeff 1932: 1562. Jeekel 1971: 313, 1984: 85, 1986: 46. Hoffman 1980: 186. Mesibov 2002: 532 (synonymised). Golovatch et al. 2009: 3. Mesibov 2009: 67.

#### Type and only species:

*Atopodesmus parvus* Chamberlin, 1920, by original designation.

#### Other included species:

*Atopodesmus allynensis* sp. n., *Atopodesmus bellendenkerensis* sp. n., *Atopodesmus carbinensis* sp. n., *Atopodesmus dorrigensis* sp. n., *Atopodesmus golovatchi* Mesibov, 2009, *Atopodesmus magnus* sp. n., *Atopodesmus minor* sp. n., *Atopodesmus otwayensis* sp. n.

#### Diagnosis.

Small Dalodesmidea (4–6 mm long as adults) with head + 19 rings; adults yellow-brown and often encrusted with soil particles, juveniles pure white and not encrusted; collum, metatergites and preanal ring with 3–6 transverse rows of small, uniform tubercles, each bearing a single seta with a slightly flared tip; ring 2 paranotum expanded, extending forward to partly cover collum edge and backward to lie under anterior edge of ring 3 paranotum; all paranota lying low on sides, flexed downward and covering legs, with a few indistinct outer marginal lobes; pore formula normal, each ozopore opening on short, columnar structure arising just dorsal to the centre of the paranotum base; legs short, without sphaerotrichomes; gonopod aperture transversely ovoid, posterior rim slightly raised; gonocoxae entirely contained within aperture, small, distally tapered, lightly joined (not fused) medially; gonopod telopodites slender, parallel and close together, more or less straight, reaching bases of legpair 4 or 5 when retracted.

#### Remarks.

The genus description I offered nine years ago (Mesibov 2002) still largely applies to *Atopodesmus golovatchi* and the seven new species described below. The only significant changes are in number of transverse rows of tubercles on midbody tergites (varying from 3–6 in the genus, rather than 5–6) and in gonopod telopodite structure, which varies considerably from species to species. An *Asphalidesmus* adult can be easily recognised by its colour, by the size and position of the ring 2 paranota, and by the characteristic dorsal tuberculation, and can be distinguished using these features alone from similar-looking Australian Pyrgodesmidae and species of *Agathodesmus* Silvestri, 1910. However, whereas each of the latter taxa has a distinctive telopodite form as well as unique non-gonopodal features, *Asphalidesmus* telopodites are remarkably dissimilar (see descriptions and illustrations below).

In particular, there does not seem to be a common location on the telopodite for the opening of the prostatic groove. In the descriptions below I have avoided using the word ‘solenomere’ for the process with this opening, because doing so might suggest that those processes are homologous across the genus, and I doubt that they are. The prostatic groove opens on anterior and posterior branches in different *Asphalidesmus* species (with no sign of torsion in the course of the groove), on the tips of processes and subapically, and on the medial and lateral sides of the telopodite.

In his review of volvatory Polydesmida, Golovatch (2003) noted that the two Tasmanian *Asphalidesmus* species known at that time had only limited ability to coil (Fig. 1C), yet both had several anatomical features found in tightly-coiling ‘oniscoid’ polydesmidans in other families: short legs, downward-flexed paranota and a slight overlap of paranota on successive rings. Three of the new *Asphalidesmus* species are known to coil tightly (Fig. 1B), although in these species the paranota are short (on anterior-posterior axis) and do not overlap.

### Asphalidesmus allynensis sp. n.

urn:lsid:zoobank.org:act:68732D56-5639-48F4-824C-676F98813319

http://species-id.net/wiki/Asphalidesmus_allynensis

**Figure 2.**
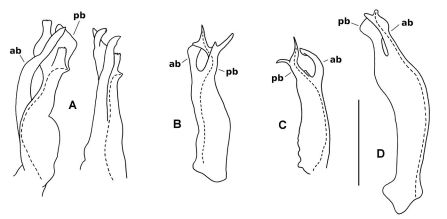
Gonopod telopodites, drawn to same scale; scale bar = 0.25 mm. Dashed lines indicate course of prostatic groove; setae not shown; **ab** = anterior branch, **pb** = posterior branch. **A** *Asphalidesmus allynensis* sp. n., holotype, AM KS94167, medial (left) and anteromedial views (right) of left gonopod telopodite. **B** *Atopodesmus dorrigensis* sp. n., paratype, AM KS61085, posterolateral view of right gonopod telopodite **C** *Atopodesmus magnus* sp .n., paratype, QM S90026, medial view of right gonopod telopodite **D** *Atopodesmus otwayensis* sp. n., paratype, ANIC 64-000207, posteromedial view of right gonopod telopodite.

Figs 2A 3A map fig. 12 

#### Holotype.

Male, Allyn Stream, Barrington Tops, NSW, “32°14'S, 151°30'E” (label data, incorrect; see Remarks), 1 February 1975, P.M. Johns, in *Nothofagus moorei* [forest], AM KS94167. Gonopods and remainder of body in two separate genitalia vials in the same sample tube.

#### Other material.

None known.

#### Diagnosis.

Gonopod telopodite branching at ca one-third telopodite height, 5 transverse rows of tubercles on midbody metatergites.

#### Description.

**Figure 3.**
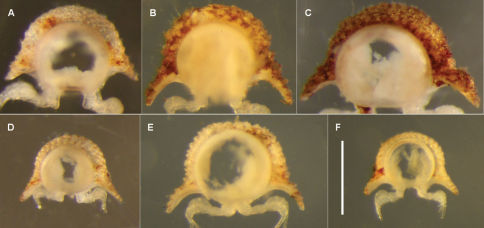
Anterior views of ring 6 (**A–D, F**) and ring 9 (**E**) of male *Asphalidesmus* spp., photographed at same scale; scale bar = 0.5 mm. **A** *Atopodesmus allynensis* sp. n., holotype, AM KS94167 **B** *Atopodesmus dorrigensis* sp. n., paratype, AM KS61085 **C** *Atopodesmus otwayensis* sp. n., paratype, ANIC 64-000207 **D** *Atopodesmus bellendenkerensis* sp. n., paratype, QM S90018 **E** *Atopodesmus carbinensis* sp. n., holotype, QM S90023 **F** *Atopodesmus minor* sp. n., paratype, ANIC 64-000205. (See Fig. 1 for comparable views of *Atopodesmus leae* Silvestri, 1910 and *Atopodesmus magnus* sp. n.)

Specimen somewhat decoloured and macerated, length ca 6 mm, ring 6 vertical diameter ca 0.6 mm and maximum width ca 0.9 mm. Midbody metatergites with 5 transverse rows of tubercles dorsally. Paranota narrow (Fig. 3A), margin clearly divided into 3 (occasionally 4) lobes.

Gonopod telopodite (Fig. 2A) divided at ca one-third telopodite height into anterior and posterior branches, below the division somewhat expanded posterolaterally, the base setose up to the division on posterior and posterolateral surfaces, the longest setae close to the division and directed distally. Posterior branch stout, curving first posteriorly, then distolaterally, mediolaterally flattening and expanding in distal half, the distal margin notched in anterior half, anterior to the notch the margin slightly bent medially and finely dentate. Posterior branch also with small, flat, near-rectangular process arising on medial surface of branch at ca one-half branch height and directed distally and slightly medially, the distal and posterior margins of the process roughened. Anterior branch more slender than posterior branch, curving smoothly posterodistally, tip slightly flattened mediolaterally and bent to lie medial to expanded tip of posterior branch and just anterodistal and lateral to tip of rectangular process on that branch. Prostatic groove following anterior surface of posterior branch, then curving posteriorly to terminate in small, conical projection on posteromedial surface of branch at origin of rectangular process.

#### Distribution.

So far known only from cool temperate rainforest on the Barrington Tops in central, near-coastal New South Wales (Fig. 12; see also Remarks).

#### Etymology.

For the Allyn River, type locality of this species.

#### Remarks.

‘Allyn Stream’ seems to be an obsolete local name for the Allyn River, whose tributaries flow through high-elevation *Nothofagus moorei* rainforest. The latitude/longitude on the printed specimen label marks the start of the Allyn River Road in long-cleared farmland at ca 220 m elevation. The latitude/longitude may have been added when the original handwritten label was replaced by a printed one (G. Milledge, pers. comm., 1 March 2011) The most likely collection sites are ca 10 km to the north-northwest, on forest roads through *Nothofagus moorei* forest above ca 700 m. My best guess is 32°09'S, 151°27'E ±3 km (see Appendix).

### Asphalidesmus bellendenkerensis sp. n.

urn:lsid:zoobank.org:act:C1B3140F-F571-4942-992F-2A5DFBC1D8B7

http://species-id.net/wiki/Asphalidesmus_bellendenkerensis

Figs 3D 4A, 4B map fig. 12 

#### Holotype.

Male, Bellenden Ker Range, Qld, cable tower 3, *17°16'04"S, 145°53'00"E*(see Remarks) ±0.25 km, 1000 m, 17–24 October 1981, Queensland Museum staff and ‘Earthwatch’ personnel, QM S90017.

#### Paratypes.

2 males, 2 females, details as for holotype but 25–31 October 1981, QM berlesate 324, rainforest, sieved litter, QM S90020; 5 males, 6 females, same details but QM berlesate 330, QM S90018; 4 males, 3 females, same details but QM berlesate 333, stick brushings, QM S90021; 1 male, 2 females, same details but summit TV station, *17°15'52"S, 145°51'14"E* (see Remarks) ±0.25 km, 1560 m, 1–7 November 1981, QM berlesate 337, QM S90019.

#### Other material.

2 males, North Bell Peak via Gordonvale, Qld, *17°05'19"S, 145°52'44"E* ±0.5 km, 900 m, 16 September 1981, G. Monteith and D. Cook, QM berlesate 300, rainforest, sieved litter and moss, QM S90022.

#### Diagnosis.

Gonopod telopodite branches curling around and nested in plane at right angles to long axis of telopodite, 4 transverse rows of tubercles on midbody metatergites.

#### Description.

Males and females approximately the same size, length ca 4 mm, ring 6 vertical diameter ca 0.4 mm and maximum width ca 0.7 mm. Midbody metatergites with 4 transverse rows of tubercles dorsally. Paranota wide (Fig. 3D); anterior and lateral margins in single convex curve, posterior margin straight; 3–4 weakly defined marginal lobes.

**Figure 4.**
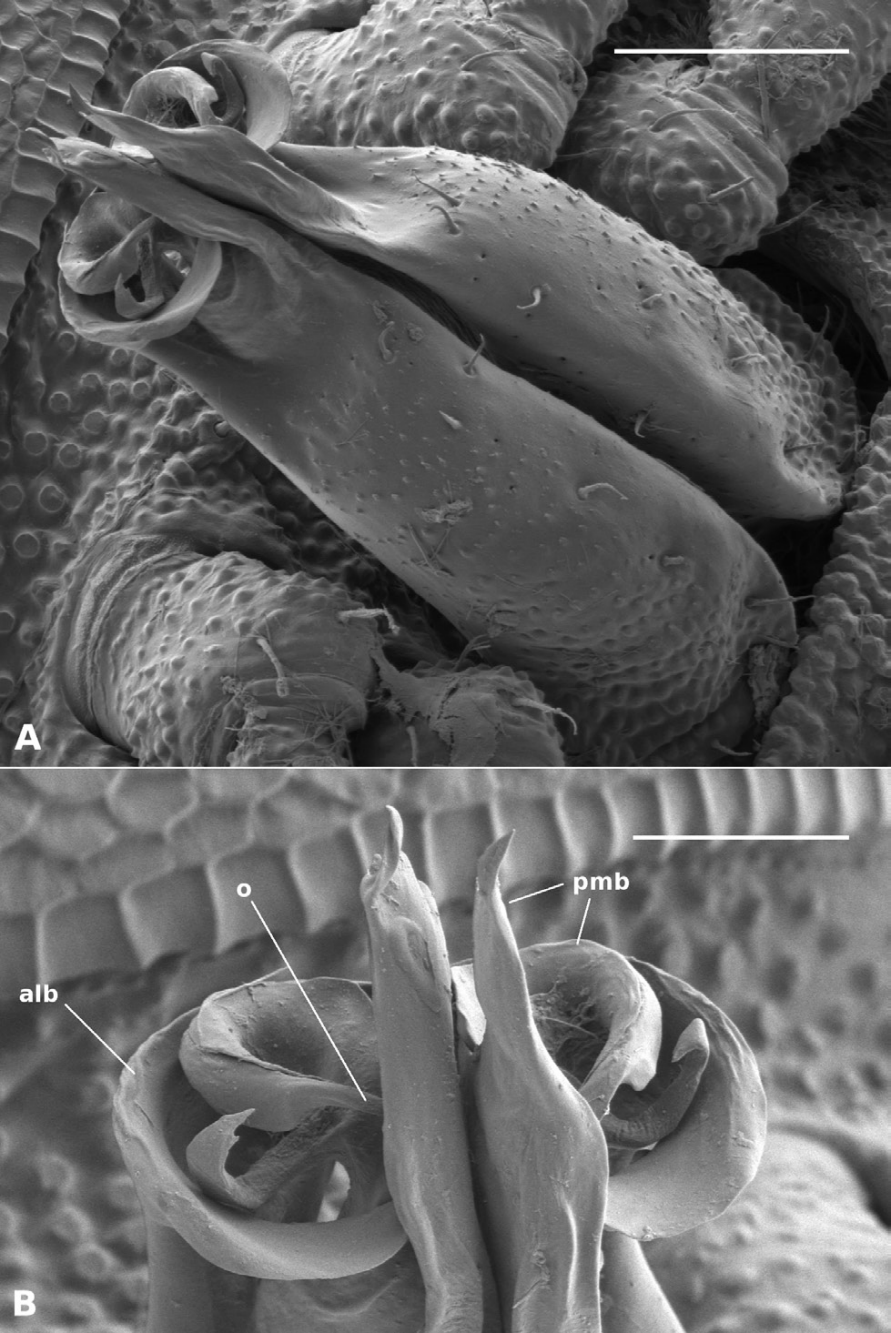
*Asphalidesmus bellendenkerensis* sp. n., paratype, QM S90018. Views of gonopods in situ (**A**) and telopodite tips (**B**). **alb** = anterolateral branch, **pmb** = posteromedial branch, **o** = points to process on which prostatic groove opens. Scale bars: **A** = 0.05 mm, **B** = 0.025 mm.

Gonopod telopodite (Figs 4A, 4B) upright, rounded-triangular in cross-section, tapering slightly and flattening distally, with small, scattered setae on posterior surface to ca two-thirds telopodite height; divided at ca seven-eighths telopodite height into complex, flattened anterolateral and posteromedial branches. Anterolateral branch with distal margin curving in plane at approximate right angle to telopodite long axis, extending in tight arc posteriorly as blade-like process terminating in fold of posteromedial process close to inner (medial) side of telopodite. Posteromedial branch folded laterally on posterior margin of process, extending distally as flat process with truncate tip, the tip bluntly dentate with taller, triangular extension at posterior end. Posteromedial branch with two additional processes: (1) anterior process nested by, and curving to follow, anterolateral branch, terminating in fold of posteromedial branch just distal to tip of anterolateral branch process; (2) small lateral process arising ca halfway across diameter of ‘circle’ formed by anterolateral branch, directed posterolaterally, curling distally at tip where it reaches anterolateral branch. Prostatic groove on anteromedial surface, abruptly entering anterior process of posteromedial branch and following arc of process, opening at process tip.

#### Distribution.

Known from tropical rainforest in far north Queensland on the Bellenden Ker Range and on the Malbon Thompson Range near Gordonvale; the two localities are ca 20 km apart (Fig. 12).

#### Etymology.

For the type locality of this species. At the summit of the range is the wettest meteorological station in Australia (Australian Bureau of Meteorology site 31141), averaging more than 8 m of rain per year. Three of the *Atopodesmus bellendenkerensis* specimens are from this site.

#### Remarks.

*Atopodesmus bellendenkerensis* is a striking exception to the dalodesmidean ‘rule of thumb’ that smaller species have simpler gonopods. The complex topology of the telopodite tip can only be clearly seen at high magnification using a scanning electron microscope (Fig. 4B).

This species can coil tightly in a spiral, but most of the specimens examined are only partly coiled.

Latitude/longitude data for the Bellenden Ker Range sites were obtained using Google Earth with advice from the Bellenden Ker cableway operator. The latitude/longitude figures for North Bell Peak are from the QM collection database.

### Asphalidesmus carbinensis sp. n.

urn:lsid:zoobank.org:act:747EE116-BC34-4F60-9BEF-05300EE8BC75

http://species-id.net/wiki/Asphalidesmus_carbinensis

Figs 3E 5A, 5B map fig. 12 

#### Holotype.

Male, Mt Lewis Road, Qld, 29 km from highway, *16°30'44"S, 145°16'10"E* ±0.5 km, 1210 m, 29 November 1997, D. Cook, QM berlesate 964, rainforest, leaf litter, QM S90023, ex QM S35904. Gonopods and remainder of body in two separate genitalia vials in the same sample tube.

#### Paratypes.

1 male, 1 female, 2 km SE of Mt Spurgeon via Mt Carbine, Qld, *16°27'17"S, 145°12'26"E* ±0.5 km, 1100 m, 20 December 1988, G. Monteith and G. Thompson, QM berlesate 825, rainforest, sieved litter, QM S90024.

#### Other material.

None known.

#### Diagnosis.

Gonopod telopodite not obviously divided into branches, instead with 4 small, pointed apical processes; 4 transverse rows of tubercles on midbody metatergites.

#### Description.

Males and females approximately the same size, length ca 5 mm, ring 6 vertical diameter ca 0.5 mm and maximum width ca 0.9 mm. Midbody metatergites with 4 transverse rows of tubercles dorsally. Paranota wide (Fig. 3E); anterior and lateral margins in single convex curve, posterior margin straight; 3–4 weakly defined marginal lobes.

**Figure 5.**
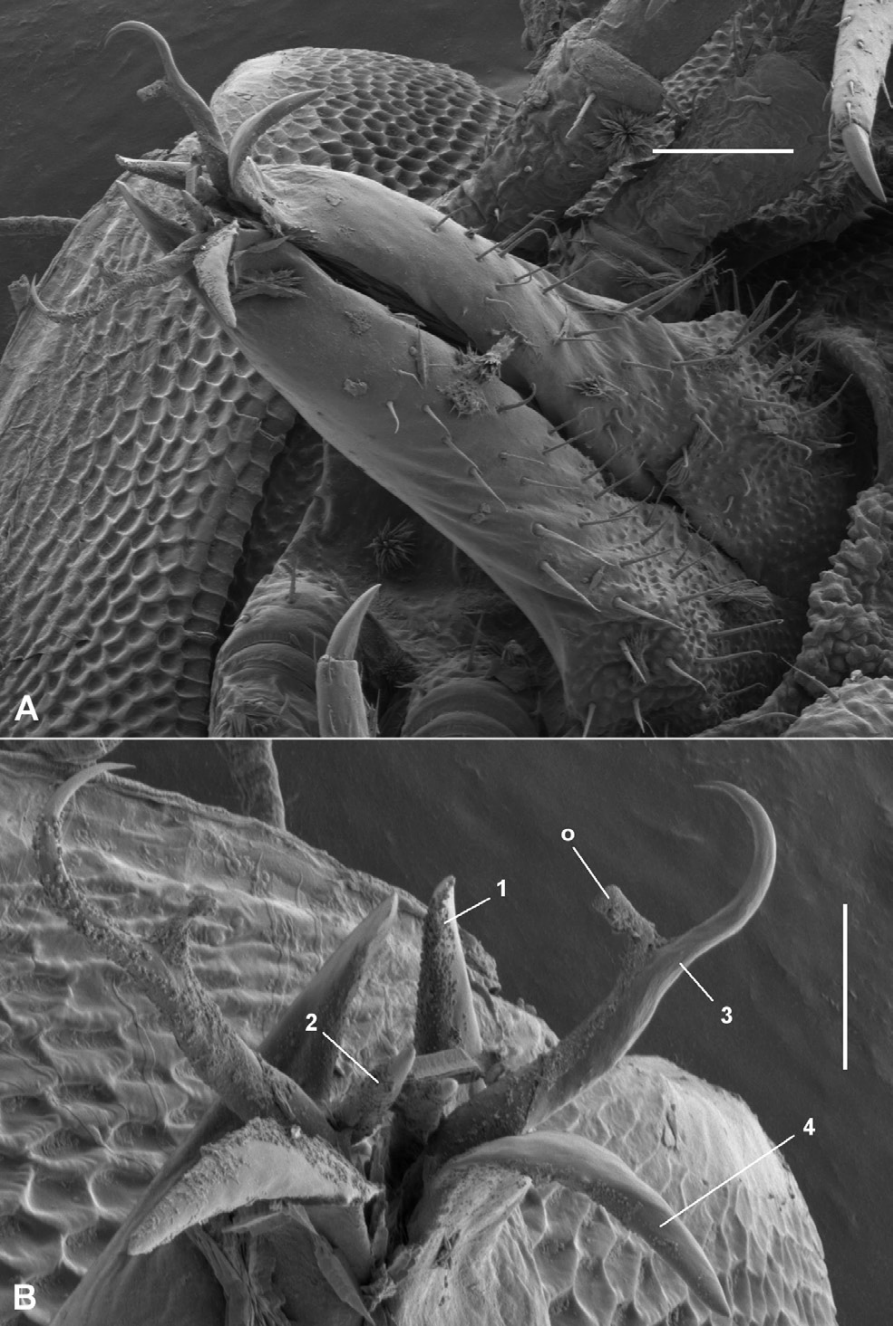
*Asphalidesmus carbinensis* sp. n., paratype, QM S90024. Views of gonopods in situ (**A**) and telopodite tips (**B**). **1, 2, 3, 4** = processes 1–4, respectively (see text for explanation), **o =** points to process on which prostatic groove opens. Scale bars: **A** = 0.05 mm, **B** = 0.025 mm.

Gonopod telopodite (Figs 5A, 5B) cylindrical, tapering distally, extended laterally at base as flange; basal one-quarter of telopodite with numerous minute, round bumps on posterior and posterolateral surfaces; sparse, strong setae to ca two-thirds telopodite height on posterior and posterolateral surfaces. Telopodite not evidently divided into branches, instead with cluster of 4 small, pointed processes at apex. From anterior to posterior: (1) short, spine-like process directed distally and very slightly medially; (2) short, spine-like process, slightly smaller than process (1), directed distally and slightly posteriorly; (3) longest process, directed distolaterally, tapering to sharp point curled medially to form ‘fish-hook’, with short, blunt, medial extension at ca two-thirds process height; (4) blade-like process, intermediate in length between (1) and (3), directed distally but bent posterolaterally at ca one-third process height. Prostatic groove on anteromedial surface of telopodite, entering process (3) and terminating on the short, blunt extension.

#### Distribution.

So far known only from tropical rainforest on the Carbine Range in far north Queensland (Fig. 12).

#### Etymology.

For the Carbine Range, type locality of this species.

#### Remarks.

Latitude/longitude data are from the QM collection database.

### Asphalidesmus dorrigensis sp. n.

urn:lsid:zoobank.org:act:9AF852C4-C859-4780-ABE2-97362164915D

http://species-id.net/wiki/Asphalidesmus_dorrigensis

Figs 1B 2B 3B 6A, 6B map fig. 12 

#### Holotype.

Male, Dorrigo National Park, NSW, west bank of Rosewood River, end of Little North Arm Road, 30°24'03"S, 152°46'18"E ±50 m, 110 m, 10–24 November 1999, M. Gray, G. Milledge and H. Smith, pitfall traps, Hotspots NE NSW site 7, AM KS114458, ex KS61085.

#### Paratypes.

7 males, 14 females, 29 adults tightly coiled and not checked for gender, details as for holotype, AM KS61085.

#### Other material.

(All from NSW) 11 males, 3 females, Dorrigo National Park, *30°22'S, 152°43'E* ±1 km, <1000 m, 7 November 1967, R.J. Bartell and L.B. Barton-Browne, ANIC berlesate 40, palm/rainforest, leaf mould, ANIC 64-000200; 6 males, 5 females, Cobcroft camp, Werrikimbe National Park, *31°15'S, 152°11'E* ±1 km, 12 November 1982, J. Doyen, ANIC berlesate 858, closed rainforest litter, ANIC 64-000201; 8 males, 7 females, Cobcroft Creek, Werrikimbe National Park, 31°16'S, 152°11'E ±1 km, 13 June 1982, L. Hill, ANIC berlesate 832, closed forest litter, ANIC 64-000202; 2 males, Horseshoe Road, Scotchman State Forest, 3.5 km SE of Thora, 30°26'25"S, 152°47'30"E ±50 m, 100 m, 10–24 November 1999, M. Gray, G. Milledge and H. Smith, pitfall traps, Hotspots NE NSW site 18, AM KS61697.

#### Diagnosis.

Gonopod telopodite with anterior branch undivided, posterior branch divided into 2 bifid processes; 3–4 transverse rows of tubercles on midbody metatergites.

#### Description.

Males slightly smaller than females, length ca 5 mm, ring 6 vertical diameter ca 0.5 mm and maximum width ca 0.9 mm. Midbody metatergites with 3–4 transverse rows of tubercles dorsally. Paranota wide (Figs 1B, 3B); anterior and lateral margins in single convex curve, posterior margin straight; 3–4 weakly defined marginal lobes.

**Figure 6.**
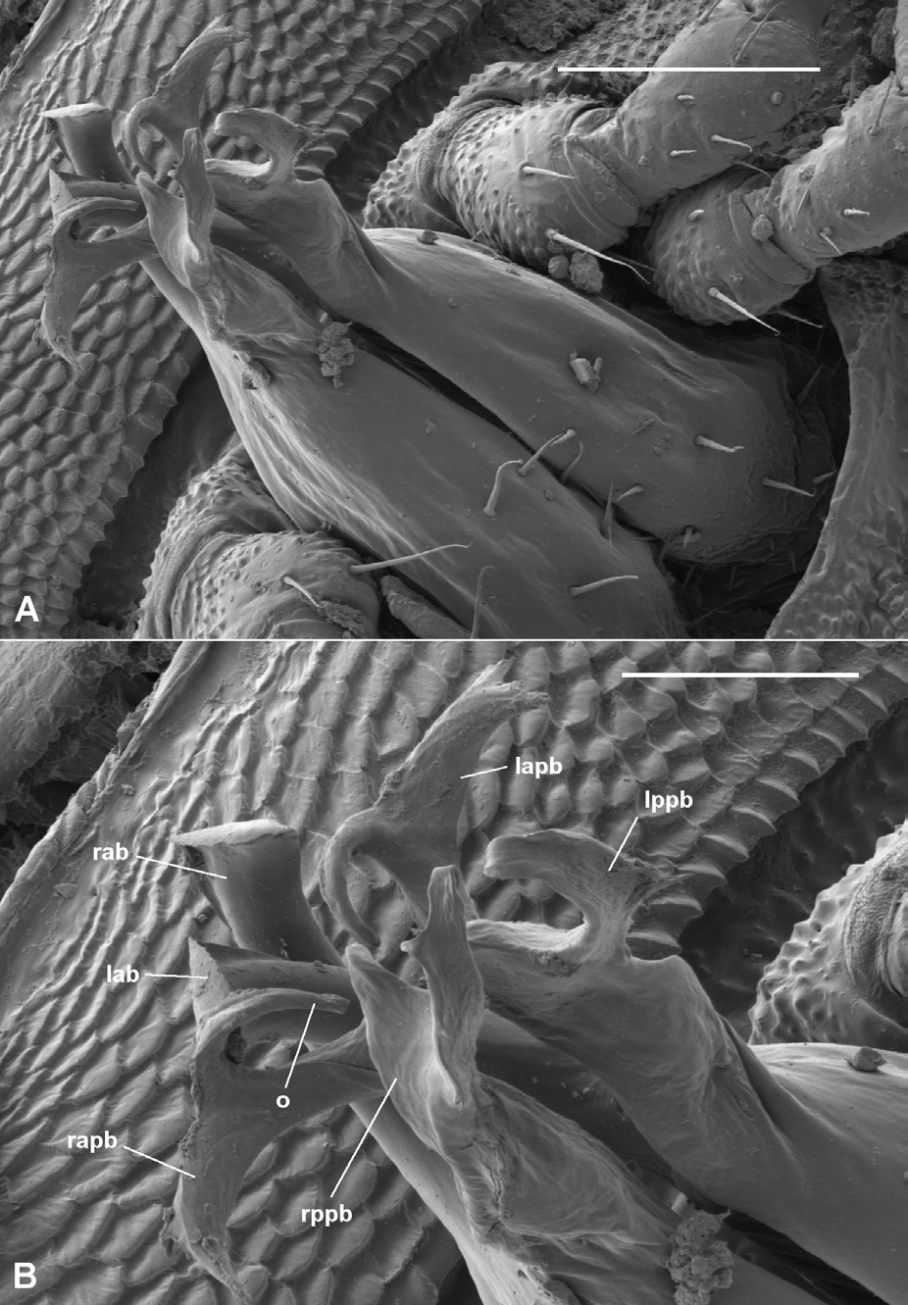
*Asphalidesmus dorrigensis* sp. n., paratype, AM KS61085. Views of gonopods in situ (**A**) and telopodite tips (**B**). **lab** = anterior branch of left gonopod (broken at base, interlaced with right gonopod branches), **rab** = anterior branch of right gonopod, **lapb** = anterior process of posterior branch of left gonopod, **rapb** = anterior process of posterior branch of right gonopod (broken at base, leaning laterally; medial arm bent over due to drying), **lppb** = posterior process of posterior branch of left gonopod (medial arm bent over due to drying), **rppb** = posterior process of posterior branch of right gonopod, **o** = points to process on which prostatic groove opens. Scale bars: **A** = 0.1 mm, **B** = 0.05 mm.

Gonopod telopodite (Figs 2B, 6A, 6B) more or less cylindrical, tapering distally, divided at between two-thirds and three-quarters telopodite height into anterior and posterior branches, a few scattered setae basally on posterior and posteromedial surfaces. Anterior branch directed anterodistally, curving slightly medially, flattening apically, tip with thin, ovoid fringe (folded over in Fig. 5B). Posterior branch divided into Y-shaped anterior and posterior processes. Posterior process directed distally, with ‘Y’ in anteroposterior plane, both arms of ‘Y’ rounded at tip. Anterior process bent slightly laterally, with ‘Y’ in mediolateral plane, medial arm directed distally (curled over in Fig. 5B), lateral arm directed laterally with small tab near tip on anterior surface. Prostatic groove on anteromedial surface of telopodite, running to medial arm of anterior process of posterior branch and terminating at tip.

#### Distribution.

Rainforest and wet eucalypt forest in northeastern, near-coastal New South Wales (Fig. 12).

#### Etymology.

For Dorrigo National Park, type locality of this species.

#### Remarks.

*Atopodesmus dorrigensis* can coil tightly in a spiral (Fig. 1B).

The branches of the gonopod seem to be fairly fragile, and are broken near the base of the branch on several of the males examined. In the specimen illustrated in Figs 6A and 6B, the contralateral branches are interlaced as a result of breakage.

Latitude/longitude data in italics are from the ANIC collection database.

### Asphalidesmus magnus sp. n.

urn:lsid:zoobank.org:act:FB9BD0E1-F23F-46E5-8422-58F4AB8C1830

http://species-id.net/wiki/Asphalidesmus_magnus

Figs 1D, 1F 2C 7 10A, 10B, 10C map fig. 12 

#### Holotype.

Male, Mt Haig, Lamb Range, Qld, *17°05'52"S, 145°36'09"E* ±0.5 km, 1000 m, 25 February 1997, G. Monteith, QM berlesate 918, rainforest, leaf litter, ex QM S37557, QM S90025.

#### Paratypes.

5 males, 5 females, details as for holotype, QM S90026.

#### Other material.

(All from Qld) 2 males, 2 females, Cammoo Caves near Rockhampton (see Distribution), 23°10'S, 150°28'E ±1 km, 25 October 1976, R.W. Taylor and T.A. Weir, ANIC berlesate 535, dense low closed forest, ANIC 64-000204; 1 male, 1 female, 3 km W by S of Mt Haig, Lamb Range, Qld, 17°06'S, 145°34'E ±1 km, 1150 m, 3 April 1984, A. Calder and T.A. Weir, ANIC berlesate 952, rainforest, ANIC 64-000203; 1 male, Lambs Head, 10 km W of Edmonton, *17°01'23"S, 145°38'33"E* ±0.5 km, 1200 m, 4 December 1988, G. Monteith and G. Thompson, QM berlesate 806, rainforest, sieved litter, QM S90028; 2 males, Vine Creek, Majors Mountain, *17°40'58"S, 145°32'02"E* ±0.5 km, 1050 m, 5 February 1999, G. Monteith and D. Cook, QM berlesate 987, rainforest, sieved litter, QM S90027.

#### Diagnosis.

Dorsum distinctly flattened anteriorly; 2 small, rounded, paramedian swellings dorsally on rings 16–18; 3 transverse rows of tubercles on midbody metatergites; gonopod telopodite with posteriorly curving anterior branch and Y-shaped posterior branch directed posterodistally and slightly laterally.

#### Description.

Males and females approximately the same size, length ca 5 mm, ring 6 vertical diameter ca 0.6 mm and maximum width ca 1.1 mm. Body strongly tapered from wide head to narrow telson (Fig. 1D); dorsum flattened anteriorly (Figs 1F, 10C); rings 16–18 with 2 small, rounded, paramedian swellings on (meta)tergites (Figs 10A, 10B). Midbody metatergites with 3 transverse rows of tubercles dorsally. Paranota wide (Fig. 1F); anterior and lateral margins in single convex curve, posterior margin straight; 3–4 weakly defined marginal lobes.

**Figure 7.**
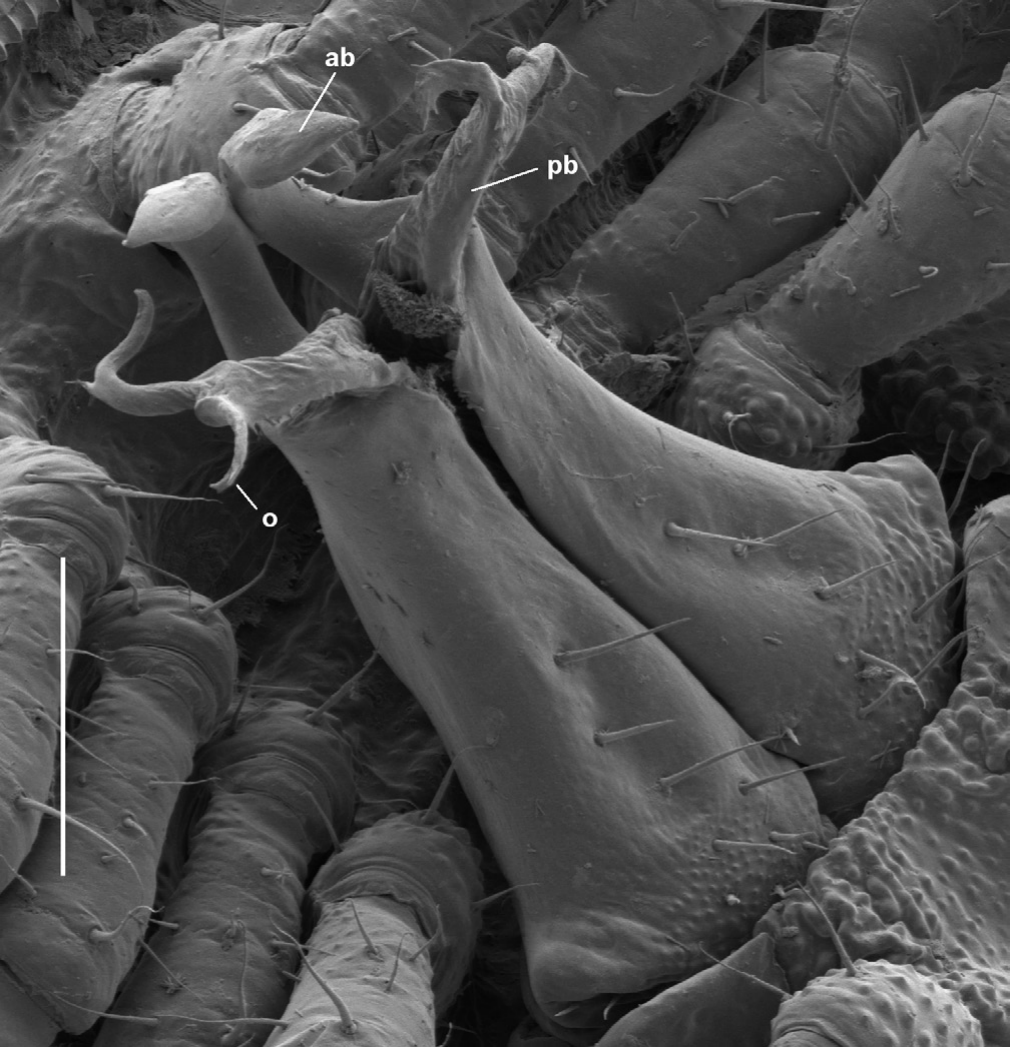
*Asphalidesmus magnus* sp. n., paratype, QM S90026, gonopods in situ. **ab** = anterior branch, **pb** = posterior branch, **o =** points to process on which prostatic groove opens. Scale bar = 0.1 mm.

Gonopod telopodite (Figs 2C, 7) more or less cylindrical, tapering distally from laterally extended base, with a few strong setae on posterior surface, divided at ca three-quarters telopodite height into anterior and posterior branches. Anterior branch somewhat flattened anteroposteriorly, with the lateral margin extended as rounded triangle basally; branch directed distally but curving posteriorly, the tip spade-like, pointed and slightly thickened. Posterior branch flattened mediolaterally, directed posterodistally and slightly laterally; branch Y-shaped, divided at ca one-half branch length into thin arms, one directed distally and the other laterally, both arms tipped with minute, variably positioned processes. Prostatic groove on anteromedial surface of telopodite, following posterior branch and terminating at tip of distally directed arm.

#### Distribution.

Known from tropical rainforest in far north Queensland and from a single collection near Cammoo Caves in central coastal Queensland (Fig. 12). Cammoo Caves are ca 840 km from the type locality of *Atopodesmus magnus*, but the four specimens from the Caves differ from the types mainly in being marginally larger; the gonopods are almost identical. If the specimens are indeed from forest near the Caves, then *Atopodesmus magnus* may have been accidentally introduced to the area from far north Queensland. This record needs to be checked by further sampling in the Cammoo Caves area.

#### Etymology.

Latin *magnus*, ‘large’, the name also containing Latin *agnus*, ‘lamb’. *Atopodesmus magnus* is the larger of the two *Asphalidesmus* species found on the Lamb Range.

#### Remarks.

*Atopodesmus magnus* can coil tightly in a spiral.

The dorsal flattening seen in this species (Figs 1F, 10C) contrasts strongly with the smoothly rounded cross-section of the type species *Atopodesmus leae* (Figs 1E, 10D). Further, limbus elements in *Atopodesmus magnus* are noticeably longer and more slender than limbus elements in the other nine *Asphalidesmus* spp. (Fig. 11) As the difference is only detectable at very high magnification, and because elements vary in length and width around the circumference of a body ring, I am reluctant to include this character state in the species diagnosis.

Latitude/longitude data in italics are from the QM collection database.

### Asphalidesmus minor sp. n.

urn:lsid:zoobank.org:act:97FB1A5F-F95E-446A-BD35-0ABBDA950274

http://species-id.net/wiki/Asphalidesmus_minor

Figs 3F 8 map fig. 12 

#### Holotype.

Male, 3 km W by S of Mt Haig, Lamb Range, Qld, 17°06'S, 145°34'E ±1 km, 1150 m, 3 April 1984, A. Calder and T.A. Weir, ANIC berlesate 952, rainforest, ANIC 64-000209.

#### Paratypes.

2 males, details as for holotype, ANIC 64-000205; 1 female, Lamb Range, 19 km S of Mareeba, *17°06'39"S, 145°34'04"E* ±0.5 km, 1200 m, 3 December 1988, G. Monteith and G. Thompson, QM berlesate 804, rainforest, sieved litter, QM S90029.

#### Other material.

None known.

#### Diagnosis.

Gonopod telopodite sharply ridged posteriorly, not obviously divided into branches, instead extending apically as thin-walled, tube-like structure; 4 transverse rows of tubercles on midbody metatergites.

#### Description.

Males and female approximately the same size, length ca 4 mm, ring 6 vertical diameter ca 0.4 mm and maximum width ca 0.6 mm. Midbody metatergites with 4 transverse rows of tubercles dorsally. Paranota wide (Fig. 3F); anterior and lateral margins in single convex curve, posterior margin straight; 4 weakly defined marginal lobes.

**Figure 8.**
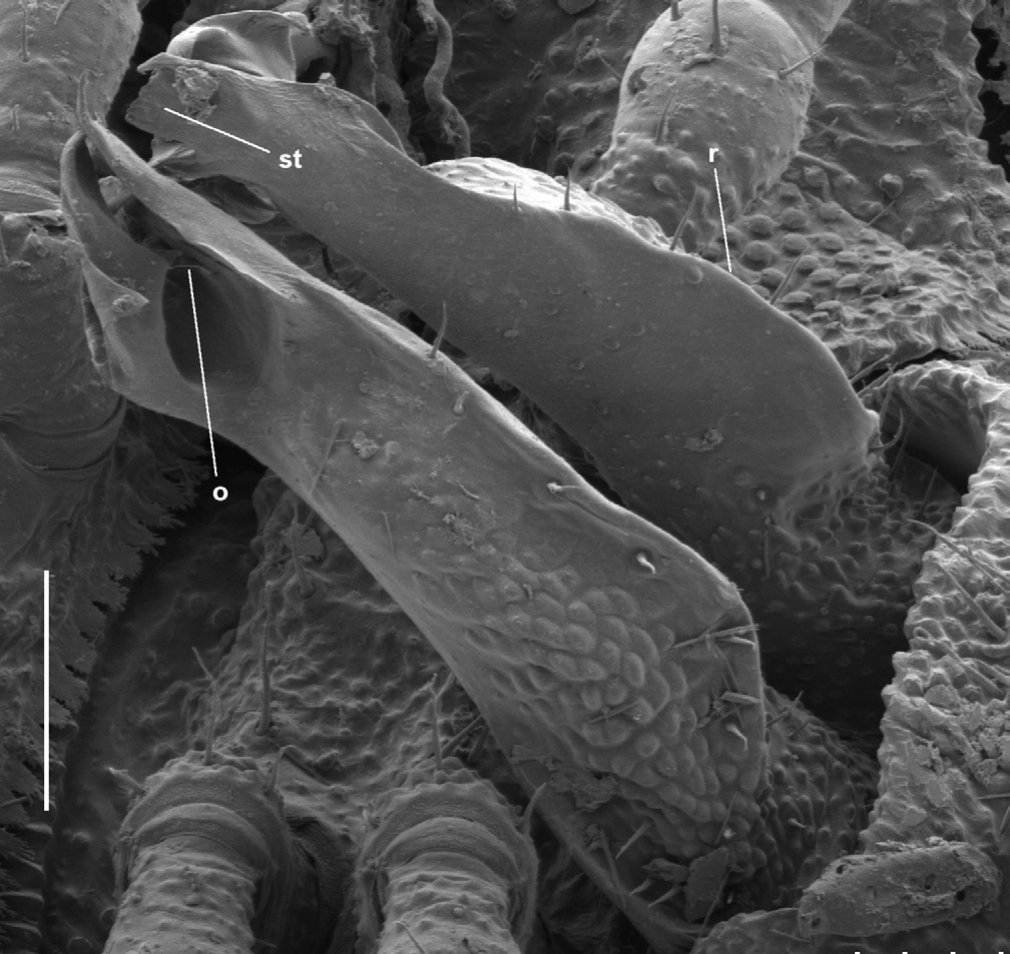
*Asphalidesmus minor* sp. n., paratype, ANIC 64-000205, gonopods in situ. **r** = posterior ridge, **st** = subquadrate tab, **o =** points to process on which prostatic groove opens. Scale bar = 0.05 mm.

Posterior surface of gonopod telopodite (Fig. 8) abruptly produced as sharp ridge from ca one-quarter telopodite height. Numerous small bumps on posterior surface basal to ridge; larger, nearly contiguous bumps lateral to ridge to ca three-quarters telopodite height; surface medial to ridge nearly smooth, flat; a few scattered setae close to ridge to ca three-quarters telopodite height. Telopodite apex extending distally as thin-walled structure with narrow posterolateral opening; apical margin extended at medial end of wall as subquadrate tab with pointed extension at its posterior end; lateral end of apical wall extended as rhomboid with rounded distal corner and rounded notch near its anterior corner. Prostatic groove on anteromedial surface of telopodite, bending abruptly at anterior end of base of subquadrate tab and directed laterally, terminating in small process projecting into space surrounded by apical wall.

#### Distribution.

So far known only from tropical rainforest on the Lamb Range in far north Queensland (Fig. 12).

#### Etymology.

Latin *minor*, ‘less’. *Atopodesmus minor* is the smaller of the two *Asphalidesmus* species found on the Lamb Range.

#### Remarks.

As with *Atopodesmus bellendenkerensis* sp. n., the topology of the telopodite apex in this tiny species is surprisingly complex, and I have not yet had a clear view under the scanning electron microscope of the tiny process carrying the opening of the prostatic groove (Fig. 8). The course of the groove before it reaches this process, however, is clearly visible under a light microscope (not shown; see Description, above).

Latitude/longitude data in italics are from the QM collection database.

### Asphalidesmus otwayensis sp. n.

urn:lsid:zoobank.org:act:A3771D42-9C7F-417E-93E8-C60C86A57D1A

http://species-id.net/wiki/Asphalidesmus_otwayensis

Figs 1G 2D 3C 9 map fig. 12 

#### Holotype.

Male, Maits Rest, Otway Ranges, Vic, *38°45'S, 143°34'E* ±1 km, 250 m, 24–25 December 1991, collector unknown (see Remarks), ANIC 64-000206.

#### Paratypes.

3 males, 2 females, details as for holotype, ANIC 64-000207.

#### Other material.

(All from Otway Ranges, Vic) 4 males, 4 females, Phillips Road, *38°39'25"S, 143°30'E* ±2 km, 25 December 1991, collector unknown (see Remarks), forest litter, ANIC 64-000208; 1 male, 1 stadium 5 female, Turtons Pass, 38°38'43"S, 143°40'36"E ±25 m, 420 m, 12 December 2003, R. Mesibov and T. Moule, MV K11142; 1 male, 1 female, same details but 38°38'39"S, 143°41'20"E ±25 m, 480 m, MV K11143; 1 male, 1 female, 1 stadium 6 female, Calder Ridge, 38°42'41"S, 143°34'03"E ±25 m, 380 m, 13 December 2003, R. Mesibov and T. Moule, MV K11141.

#### Diagnosis.

Gonopod telopodites crossed at ca two-thirds telopodite height; apodous ring 18 produced dorsally as large, rounded swelling; 5 transverse rows of tubercles on midbody metatergites.

#### Description.

Males and females approximately the same size, length ca 6 mm, ring 6 vertical diameter ca 0.6 mm and maximum width ca 1.1 mm. Midbody metatergites with 5 transverse rows of tubercles dorsally. Paranota wide (Fig. 3C); anterior and lateral margins in single convex curve, posterior margin straight with small round tab near base; 4–5 weakly defined marginal lobes. Ring 18 produced dorsally as large, rounded swelling (Fig. 1G) in both males and females.

**Figure 9.**
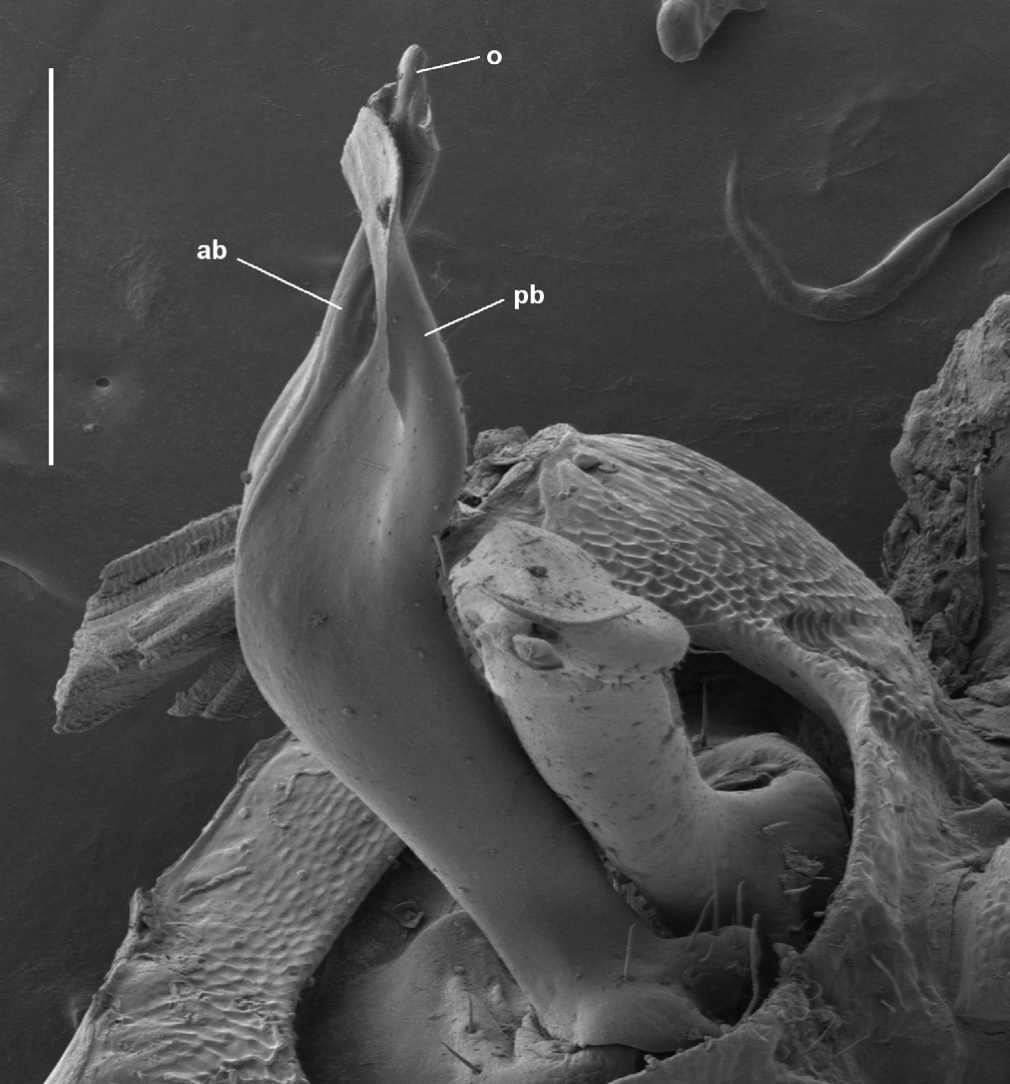
*Asphalidesmus otwayensis* sp. n., paratype, ANIC 64-000207, gonopods in situ. Right gonopod telopodite is crossed anterior to left; view of left gonopod telopodite is down its long axis. **ab** = anterior branch, **pb** = posterior branch, **o =** points to process on which prostatic groove opens. Scale bar = 0.2 mm.

Gonopod telopodite (Figs 2D, 9) divided into anterior and posterior branches at ca three-quarters telopodite height; telopodite widest just below division, bent posterodistally at ca one-half telopodite height, tapering basally to small base with small, rounded lobes directed posteriorly and posterolaterally, each lobe carrying a few small setae; a few small setae on posterior surface of telopodite just basal to division. Anterior branch anteroposteriorly flattened, directed posterodistally, expanded at tip into rhomboid, the central portion of the rhomboid thickened as finger-like process. Posterior branch similarly flattened, directed distally, the tip expanded into rhomboid lying just anterior to and parallel with rhomboid of anterior branch (leaving a small gap between), thus crossing anterior branch and terminating anterior to the latter’s tip. Prostatic groove on anterior surface of telopodite, continuing on anterior branch to terminate at tip of finger-like process. Gonopod telopodites crossed near tips (see Remarks), with one telopodite nesting in bend on posterior surface of other telopodite.

**Figure 10.**
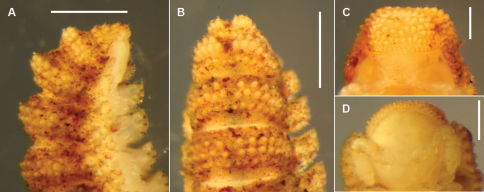
**A, B** Lateral and dorsal views of last rings of paratype male *Asphalidesmus magnus* sp. n., QM S90026 **C** Ventral and slightly frontal view of head of paratype male *Atopodesmus magnus* sp. n., QM S90026 **D** Ventral view of head of *Atopodesmus leae* Silvestri, 1910, QVM 23:41547. Scale bars: **A, B** = 0.5 mm, **C, D** = 0.25 mm.

**Figure 11.**
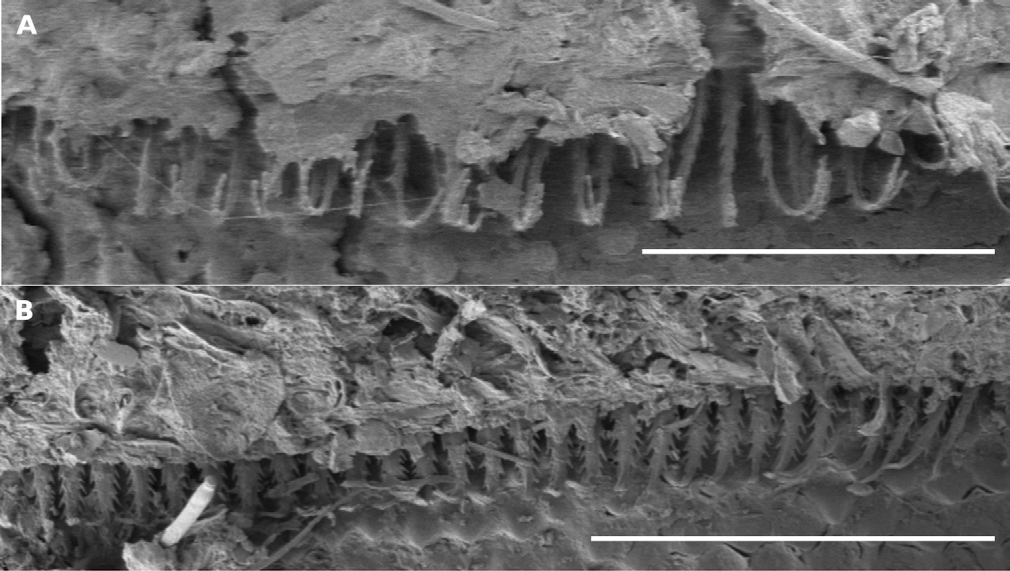
**A, B** Views of limbus on midbody metatergites; scale bars = 0.1 mm. **A** *Asphalidesmus magnus* sp. n., paratype male, QM S90026. **B** *Atopodesmus otwayensis* sp. n., ANIC 64-000208. The form of the limbus elements in *Atopodesmus otwayensis* sp. n. is typical for the genus, but as in all *Asphalidesmus* spp. the length and width of the elements varies around the circumference of a ring.

#### Distribution.

**Figure 12.**
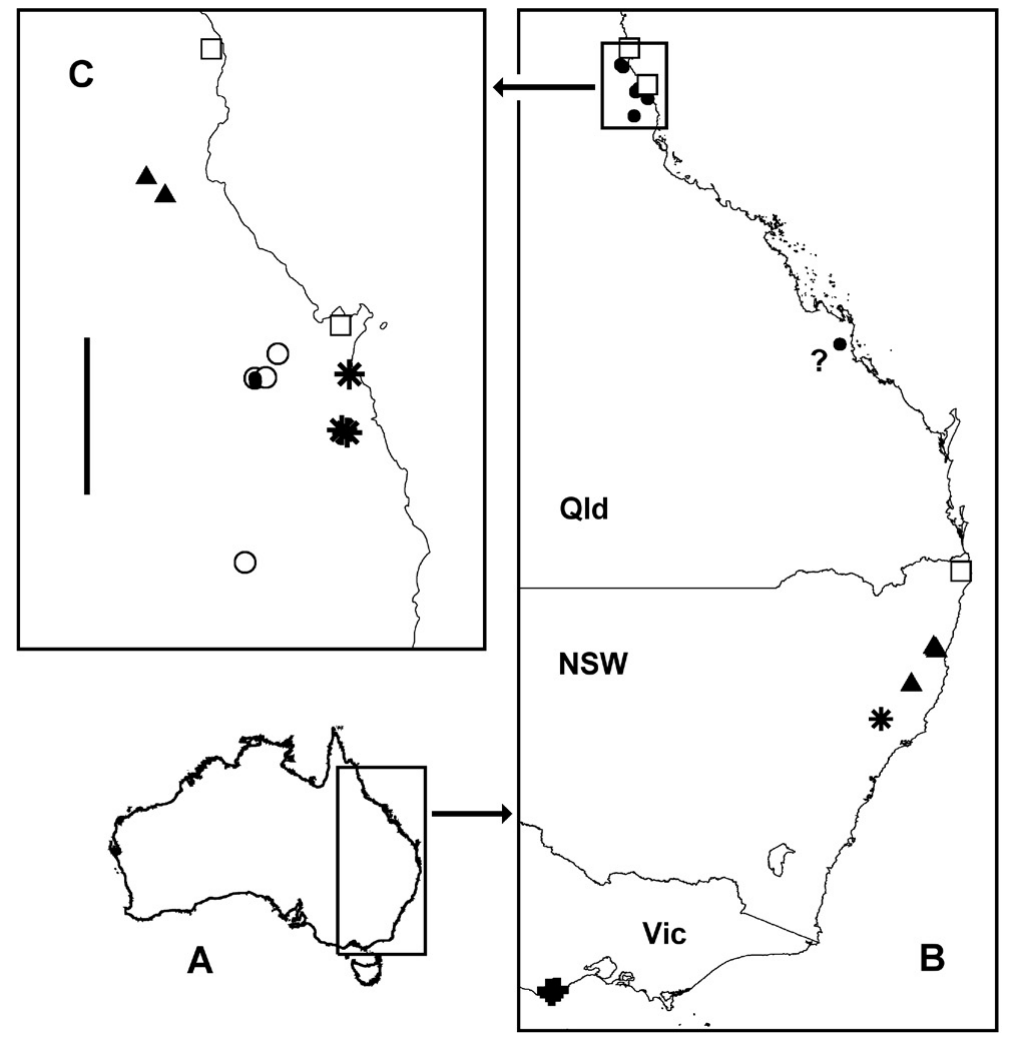
**A** Outline map of Australia showing location of map **B**. **B** Localities for *Asphalidesmus otwayensis* sp. n. (crosses), *Atopodesmus allynensis* sp. n. (star), *Atopodesmus dorrigensis* sp. n. (filled triangles), all Queensland species (filled circles), unidentified *Asphalidesmus* (open squares) and questioned locality for *Atopodesmus magnus* sp. n. (question mark). Rectangle at top shows location of map **C**. **C** Localities in far north Queensland for *Atopodesmus bellendenkerensis* sp. n. (stars), *Atopodesmus carbinensis* sp. n. (filled triangles), *Atopodesmus magnus* sp. n. (open circles), *Atopodesmus minor* sp. n. (filled circles) and unidentified *Asphalidesmus* (open squares). Scale bar = 50 km. **A** and **B** are geographic projections, **C** is Mercator projection.

Known from cool temperate rainforest and wet eucalypt forest in the Otway Ranges in southwestern Victoria (Fig. 12).

#### Etymology.

For the Otway Ranges, home to this species.

#### Remarks.

The collector of the Maits Rest and Phillips Road samples from December 1991 is not named on the ANIC sample labels, which I copied when labelling specimens sorted from ANIC mixed holdings. Museum Victoria personnel collected at Otway Ranges sites at various times between October 1991 and March 1992, but there are no MV samples from 24 or 25 December 1991 (K. Walker, pers. comm., 4 March 2011). The ANIC database also has no collection records for other taxa from these places and dates (B. Mantle, pers. comm., 4 March 2011). The most likely possibility is that an entomological collector, not from MV, coincidentally sampled in the Otway Ranges during the 1991 Christmas holiday period and deposited the material in ANIC.

Latitude/longitude data in italics are based on local maps and Google Earth.

Crossing of gonopod telopodites (Fig. 9) is unusual in Dalodesmidea. In this species it may be facilitated by the remarkably small telopodite base articulating with the gonocoxa. The right telopodite crosses anterior to the left telopodite in six of the 11 *Atopodesmus otwayensis* males examined, and posterior to the left telopodite in the other five.

Neither of the two juvenile females has a dorsal swelling on an apodous ring (ring 17 in stadium 6, rings 15 and 16 in stadium 5).

## Supplementary Material

XML Treatment for Asphalidesmus

XML Treatment for Asphalidesmus allynensis

XML Treatment for Asphalidesmus bellendenkerensis

XML Treatment for Asphalidesmus carbinensis

XML Treatment for Asphalidesmus dorrigensis

XML Treatment for Asphalidesmus magnus

XML Treatment for Asphalidesmus minor

XML Treatment for Asphalidesmus otwayensis

## Appendix I

Specimen records for the 10 currently known species of *Asphalidesmus* Silvestri, 1910.

### Note:

Specimen records for *Asphalidesmus* species are available on the *ZooKeys* website as a Microsoft Excel file (Appendix.xls, doi:10.3897/zookeys.93.1255.app.1a) and a CSV file (Appendix.csv, doi:10.3897/zookeys.93.1255.app.1b).
